# hHGF Overexpression in Myoblast Sheets Enhances Their Angiogenic Potential in Rat Chronic Heart Failure

**DOI:** 10.1371/journal.pone.0019161

**Published:** 2011-04-26

**Authors:** Antti Siltanen, Katsukiyo Kitabayashi, Päivi Lakkisto, Johanna Mäkelä, Tommi Pätilä, Masamichi Ono, Ilkka Tikkanen, Yoshiki Sawa, Esko Kankuri, Ari Harjula

**Affiliations:** 1 Institute of Biomedicine, University of Helsinki, Helsinki, Finland; 2 Department of Cardiovascular Surgery, Osaka University Graduate School of Medicine, Osaka, Japan; 3 Minerva Institute for Medical Research, Helsinki, Finland; 4 Department of Clinical Chemistry, Helsinki University Central Hospital, Helsinki, Finland; 5 Department of Cardiothoracic Surgery, Helsinki University Meilahti Hospital, Helsinki, Finland; 6 Department of Medicine, Helsinki University Central Hospital, Helsinki, Finland; University of Padova, Medical School, Italy

## Abstract

After severe myocardial infarction (MI), heart failure results from ischemia, fibrosis, and remodeling. A promising therapy to enhance cardiac function and induce therapeutic angiogenesis via a paracrine mechanism in MI is myoblast sheet transplantation. We hypothesized that in a rat model of MI-induced chronic heart failure, this therapy could be further improved by overexpression of the antiapoptotic, antifibrotic, and proangiogenic hepatocyte growth factor (HGF) in the myoblast sheets. We studied the ability of wild type (L6-WT) and human HGF-expressing (L6-HGF) L6 myoblast sheet-derived paracrine factors to stimulate cardiomyocyte, endothelial cell, or smooth muscle cell migration in culture. Further, we studied the autocrine effect of hHGF-expression on myoblast gene expression profiles by use of microarray analysis. We induced MI in Wistar rats by left anterior descending coronary artery (LAD) ligation and allowed heart failure to develop for 4 weeks. Thereafter, we administered L6-WT (n = 15) or L6-HGF (n = 16) myoblast sheet therapy. Control rats (n = 13) underwent LAD ligation and rethoracotomy without therapy, and five rats underwent a sham operation in both surgeries. We evaluated cardiac function with echocardiography at 2 and 4 weeks after therapy, and analyzed cardiac angiogenesis and left ventricular architecture from histological sections at 4 weeks. Paracrine mediators from L6-HGF myoblast sheets effectively induced migration of cardiac endothelial and smooth muscle cells but not cardiomyocytes. Microarray data revealed that hHGF-expression modulated myoblast gene expression. *In vivo*, L6-HGF sheet therapy effectively stimulated angiogenesis in the infarcted and non-infarcted areas. Both L6-WT and L6-HGF therapies enhanced cardiac function and inhibited remodeling in a similar fashion. In conclusion, L6-HGF therapy effectively induced angiogenesis in the chronically failing heart. Cardiac function, however, was not further enhanced by hHGF expression.

## Introduction

The quiescent post-inflammatory scar tissue after myocardial infarction (MI) impairs cardiac function and restricts ventricular dilatation. In a chronic situation, the active processes such as inflammation, cell death and necrosis, proteolytic activity, and overall tissue response to ischemia have gradually ended, and the tissue has become fibrotic, quiescent, and dysfunctional. In chronic heart failure (HF), regeneration of such fibroblast- and collagen-rich scar tissue depleted of myocytes has proved to be an extensive task for pharmacological or cellular therapy.

Hepatocyte growth factor (HGF) is a pleiotropic cytokine that induces mitogenesis, motogenesis, and morphogenesis [1]. In the heart, acute myocardial infarction [2], ischemia reperfusion injury [3], and congestive heart failure [4] induce expression of HGF. In myocardial ischemia, HGF has been suggested to counteract damage and to mediate a regenerative response [5]. Extensive research has focused on the beneficial effect of HGF in the acute early stages of MI; little is known, however, about the role HGF plays in the chronic stage of HF.

Cell therapy has shown promise as an alternative to heart transplantation [6]. Furthermore, transplantation of cells such as myoblasts as epicardially deposited sheets has proven a method of cell delivery superior to invasive intramyocardial injections [7], [8]. We recently showed that the effect of myoblast sheets can be further enhanced by gene therapy, and that after MI, in both acute and chronic HF their therapeutic effect is mainly mediated through production of paracrine mediators [9], [10]. Moreover, our results showed that preventing Bcl-2-dependent cell death can prolong and intensify this production of paracrine effectors from the sheets. We also identified the factors released from the sheets and demonstrated that their prolonged production induced angiogenesis in the ischemic myocardium, inhibited fibrosis, reduced ventricular dilation, and enhanced cardiac function [9]. We demonstrated that the Bcl-2-expressing myoblast sheets secreted increased amounts of vascular endothelial growth factor (VEGF) and placental growth factor (PlGF) than did the wild type sheets, and that the angiogenic responses stimulated by myoblast sheets were mediated via the Flk1/Flt1 signaling pathway [9], [10]. In addition to the VEGF family of cytokines, HGF has been demonstrated after MI to reduce fibrosis and ventricular remodeling [5], and to enhance angiogenesis [11]. Intriguingly, these are the same characteristics as were identified to be the important therapeutic components of myoblast sheet therapy [9]. Such common characteristic mechanisms suggested to us that synergism could exist between myoblast sheets and *hgf* gene therapy in treatment of HF.

In this study, our aim was to evaluate the therapeutic value of HGF production from epicardially deposited myoblast sheets in chronic HF. We hypothesized that enhancing the production of HGF from myoblast sheets would induce a synergistic therapeutic effect between the overexpressed HGF and the other cytokines, such as VEGF and PlGF, released from myoblast sheets. Moreover, HGF therapy might help reduce remodeling and fibrosis as well as increase blood flow to improve graft survival and functionality.

## Materials and Methods

### Cell culture and sheets

Myoblast cell culturing and sheet fabrication followed the method previously described [9]. The L6 rat skeletal myoblast cell line came from the American Type Culture Collection (CRL-1458, Manassas, VA) with cells at passages 5 to 15 used for experiments. We engineered myoblast cell sheets by plating 6×10^6^ myoblasts on thermoreactive cell culture dishes (CellSeed, Tokyo, Japan) for 16 hours. Intact myoblast sheets detached spontaneously from culture dishes at room temperature and were harvested for transplantation. To study the effect of paracrine mediators secreted by myoblast sheets, we washed the sheets thoroughly with serum free medium, incubated the sheets in that medium for 24 hours, and collected the conditioned medium for experiments.

To establish cultures that contain all major cell types of the myocardium, hearts of fetal Wistar rats (E17.5) were excised and underwent mincing and enzymatic digestion with trypsin (Sigma-Aldrich, Saint Louis, MO, USA) and collagenase IV (Worthington Biomedical, Lakewood, NJ). After a 30-minute enzyme digestion with shaking in a water bath at 37°C, the supernatant with cells was collected, and the remaining minced tissue was subjected to another digestion. We repeated this cycle four times until all tissue was digested. After digestion, we plated the collected supernatants in DMEM containing 10% fetal bovine serum, 5% horse serum, and antibiotics to 24-well cell culture dishes pretreated with 0.2% gelatin (Sigma-Aldrich) to promote cell adherence.

To establish cultures of cardiac fibroblasts, the myocardial cell suspensions after enzymatic digestion were plated for 90 minutes on culture dishes to allow attachment of non-myocyte cells. After this incubation, the non-adhered cell population was removed. The cultures were extensively washed to ascertain removal of myocytes from the culture. We then passaged this early-adherent cell population 6 times to allow overgrowth and enrichment of cardiac fibroblasts. We then plated these cells to 24-well plates for migration experiments or to 96-well plates for the fibrosis assay.

### hHGF transfection and verification of overexpression

We transfected the L6 myoblasts for 24 hours in the presence of pBabepuro retroviral vector and 8 µg/ml polybrene (Sigma-Aldrich) to create a cell line with constitutive overexpression of human *hgf*. The vector came from Biomedicum Genomics, Helsinki, Finland. We selected the transfected cells with incubation in growth medium containing 2 µg/ml puromycin for 48 hours. To verify the success of the transfection, we performed *in situ* hybridization of the human *hgf* mRNA in L6 myoblasts using the Ventana Discovery Automate (Ventana Medical Systems Inc, Tuczon, AZ, USA). We used the antisense primer sequence 5′-ATTTAGGTGACACTATACACAAGCAATCCAGAGGTACGC-3′ to detect hHGF mRNA and sense primer sequence 5′-TAATACGACTCACTATAGGCCTCGGCTGGCCATCGGG-3′ as the control sequence. For detection of secreted hHGF from the L6 myoblast sheet culture medium, we used the human HGF Duoset ELISA kit according to the manufacturer's protocol (R&D Systems, Minneapolis, MN).

### Analysis of cardiac cell migration

After plating, we incubated the cardiac cell cultures for 48 hours to allow proper attachment and changed the medium to serum free for a period of 24 hours. After serum deprivation, we washed the cultures and scratch-wounded them with a pipette tip. To determine the ability of myoblast sheet-derived paracrine factors and transfected *hgf* to promote migration of cardiac cells, we substituted the serum free DMEM with 24-hour conditioned medium derived from L6-WT or L6-HGF myoblast sheets. 24 hours later, we fixed the cultures with 4% paraformaldehyde and perfused the cells with Triton-X. We used immunofluorescence staining for von Willebrand factor (vWF, rabbit polyclonal, Millipore, Billerica, MA, USA) and alfa-smooth muscle actin (SMA, mouse monoclonal, DAKO Cytomation, Glostrup, Denmark) to identify and evaluate migrating endothelial and smooth muscle cells. Secondary antibodies were anti-mouse Alexa Fluor 488 and anti-donkey Alexa fluor 596 (Molecular Probes, Eugene, OR). We acquired imuunofluorescence images of the denuded area with a Olympus IX81 microscope, DP30BW camera, and Cell F 2.7 software (Olympus, Tokyo, Japan). We evaluated the number of vWF- and SMA-positive cells migrating into the denuded area with Photoshop 7.0 (Adobe Systems Inc., Delaware, CA). We acquired phase contrast images from the cardiac fibroblast cultures and evaluated their migration with Photoshop 7.0.

### Analysis of ventricle dilatation

We analyzed the dilation of the left and right ventricles from histological sections to accurately determine whether myoblast sheets can prevent MI-induced remodeling and whether *hgf* gene therapy can augment the anti-remodeling effect. We used ImageJ software (U. S. National Institutes of Health, Bethesda, MD, http://imagej.nih.gov/ij) to determine the circumference of the ventricles from hematoxylin/eosin-stained paraffin-embedded sections. Papillary muscles were omitted from the analysis.

### Animals

We operated on 58 Wistar rats (250–400 g). Of these, 49 (84.5%) survived both surgeries and were randomly divided into four groups: the control group underwent left anterior descending coronary artery (LAD) ligation and re-thoracotomy (n = 13), the L6 wild type group (L6-WT) underwent LAD ligation and sheet transplantation (n = 15), the *hgf* gene therapy group (L6-HGF) underwent LAD ligation and sheet transplantation (n = 16), and the sham operation group (n = 5) underwent thoracotomy twice. We induced MI by LAD ligation as described previously [12]. We intubated the animals and maintained respiration with a ventilator during surgery. We exteriorized the heart rapidly through a left thoracotomy and pericardiotomy and ligated the LAD 3 mm from its origin. After ligation, we returned the heart to its normal position, and covered it with pericardium to avoid adhesion to the lung and to the chest wall.

Four weeks after ligation of the LAD, all animals underwent re-thoracotomy. For animals in L6-WT and L6-HGF groups, two myoblast sheets each were transplanted on to the left ventricular anterior wall. Thus, every animal in these groups was grafted with a total of 1.2×10^7^ cells. All animals were euthanized at 4 weeks after the second surgery.

After the surgery, we antagonized anesthesia with atipamezole hydrochloride (1.0 mg/kg s.c., Antisedan®, Orion Pharma Inc, Turku, Finland) and administered buprenorphine hydrocholoride (0.05 mg/kg sc, Temgesic®, Reckitt and Colman Ltd, Hull, UK) for post-operative analgesia. Experimental procedures were conducted according to the US National Institutes of Health Guide for the Care and Use of Laboratory Animals, and were approved by the ethics committee of the HUS/Meilahti Hospital Department of Surgery (permit numbers: ESLH-2008-05359/Ym-23, STH420A and ESLH-2008-10408/Ym-23, STH991A).

### Echocardiography

All animals underwent echocardiography under anesthesia one day before the first surgery (baseline) as well as 2 weeks and 4 weeks after the second surgery. The echocardiographic measurements were performed with a 7.5 MHz transducer (MyLab®25, Esaote SpA, Genoa, Italy). We measured anterior and posterior wall thickness in the diastolic phase (AWTd, PWTd), and left ventricular diameter in both the diastolic (LVDd) and systolic (LVDs) phases in the short-axis right parasternal projection just below the mitral valves. Data was collected from three systolic cycles and averaged. We used LVDd and LVDs to calculate left ventricular fraction shortening (LVFS) and ejection fraction (LVEF) by the following formulas:
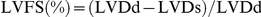


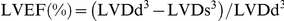



### Histology and immunostaining

Four weeks after the second surgery, and after assessment of cardiac function, all rats were euthanized. We excised the heart and cut it into four equal transverse parts. The two middle parts were fixed in 4% neutral-buffered formalin for 48 hours, embedded in paraffin, and cut into 4-µm-thick sections for histology and immunostaining.

We performed immunohistochemistry with a Ventana Discovery Automate (Ventana). Cell proliferation was evaluated by use of anti-Ki67 antibody (RM-9106, Labvision Inc, Fremont, CA). The sections stained for Ki67 proliferation-associated antigen were double-stained for myocytes with an anti-tropomyosin antibody (MS-1256, Labvision Inc). We analyzed six fields from a single section (two images from the infarct area, border area, and remote area). To analyze capillaries and arteries in the myocardium, we stained endothelial cells with a primary antibody for vWF and SMA and secondary fluorescent antibody (AlexaFluor 488 and 596). The specificity of the anti-vWF antibody for endothelial cells was proved against staining and signal co-localization with anti-PECAM-1 antibody (M-20, Santa Cruz Biotechnology, Santa Cruz, CA). We acquired images by fluorescent microscopy (Olympus). We evaluated the proportion of positive cells with ImageJ software.

Amount of fibrosis was evaluated from Sirius Red-stained paraffin-embedded sections. The Sirius Red area was divided by the whole section area to acquire a relative count of cardiac fibrosis. For analysis, we used scanned images of stained tissue sections and evaluated fibrotic area with Photoshop 7.0 (Adobe Systems Inc.).

### *In vitro* fibrosis and apoptosis assays

To study whether paracrine factors derived from L6-HGF myoblasts can inhibit collagen deposition, such as in the case of acute MI, or degrade collagen already deposited by fibroblasts as in chronic MI, we employed an *in vitro* fibrosis model. To this end, we cultured human dermal fibroblasts (ATCC, CRL-2088) or rat cardiac fibroblasts in DMEM containing 10% FBS, as detailed above, to confluency. L-ascorbic acid 2-phosphate (50 µg/ml, Sigma-Aldrich) was used to induce collagen deposition while untreated wells served as baseline. To test the ability of hHGF to inhibit collagen deposition in these cultures, we treated them with standard culture medium (control) or L6-HGF conditioned medium. Medium was changed every 2 days. At 7 days, the cultures were fixed using 10% formalin, and were washed with PBS. Deposited collagen was stained with Sirius Red-saturated in picric acid solution (Sigma-Aldrich). Cultures were then washed thoroughly with 0.5% acetic acid to remove any unbound dye. Collagen-bound Sirius Red was dissolved in 0.1 M NaOH and the amount of collagen was determined by measuring optical density at 540 nm. For the collagen degradation assay (mimicking an already formed fibrotic scar), collagen was allowed to accumulate for 7 days (baseline). Ascorbic acid was then removed from cultures and incubation was continued with control or with L6-HGF conditioned medium for another 7 days. Amount of collagen was then quantitated as described above.

To evaluate whether the hHGF secreted by the myoblasts could inhibit myoblast apoptosis in an autocrine manner and thus prolong the duration of therapy, we plated 10,000 myoblasts per well on a 96-well plate, allowed the cells to adhere for 24 hours. We then treated the cells with staurosporine to induce apoptosis for another 24 hours. Myoblast viability was quantitated by the amount of formazan dye converted from MTT by mitochondria. We dissolved the formazan dye in DMSO and measured optical density at 540 nm using a reference wavelength of 620 nm.

### Statistical analysis

All data are presented as mean ± SEM. Differences between groups were compared with ANOVA followed by a Bonferroni post test. Statistical analysis was with Graph Pad Prism 4.0 (GraphPad Software Inc., San Diego, CA).

### Analysis of gene expression

To study the effect of constitutive hHGF expression on the phenotype of the donor cells, we determined the whole genome gene expression profiles of wild type and hHGF-transfected myoblasts as monolayers or sheets. We isolated total RNA with Trizol reagent, following the manufacturers' protocol. Further sample processing and hybridization to the Affymetrix GeneChip Rat Genome 230 2.0 chip (Affymetrix, Santa Clara, CA) was by Biomedicum Genomics, University of Helsinki (http://www.biomedicumgenomics.fi). All data are complient with the Minumum Information about a Microarray Experiment (MIAME) giudelines (http://www.mged.org/Workgroups/MIAME/miame.html), and the raw and normalized data are deposited in the National Center for Biotechnology Information Gene Expression Omnibus (NCBI, GEO, http://www.ncbi.nlm.nih.gov/geo/, series accession number GSE25752) database. Data were normalized to median with the RMA algorithm.

## Results

### hHGF transfection and functionality

**Figure 1:**
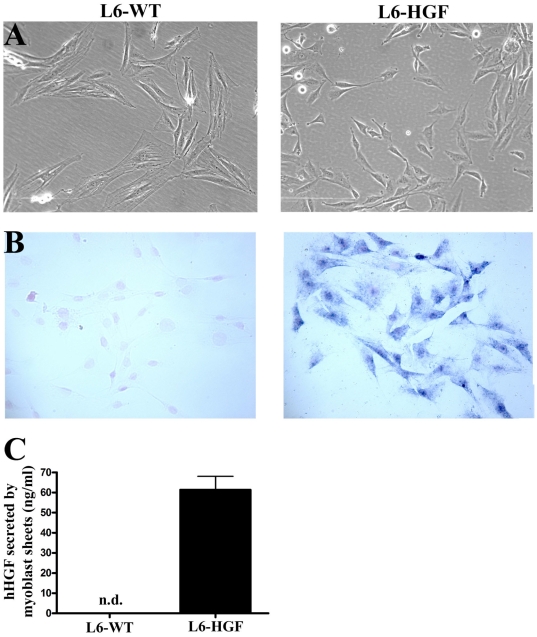
Characterization of hHGF transfection. (A) Phase contrast images representing morphology of wild type (L6-WT) or hHGF-expressing (L6-HGF) rat L6 myoblasts (B). hHGF mRNA undetectable in L6-WT myoblasts by in situ hybridization, whereas in the retrovirus-tranfected L6-HGF myoblasts, its expression was evident. Images in A and B are from different locations. (C) hHGF secreted by L6-WT and L6-HGF myoblast sheets into the culture medium in 24 hours was determined by ELISA. hHGF was undetectable from L6-WT myoblast sheets, whereas abundant secretion was detectable from L6-HGF myoblast sheets.

We used in situ hybridization to demonstrate the efficacy of hHGF transfection. In L6-HGF cells the expression of hHGF was clearly evident as compared to no detectable reactivity for hHGF mRNA in L6-WT myoblasts (Figure 1A and B).

We then evaluated the amount of hHGF protein secreted into the culture medium from the L6-HGF myoblast sheets. These sheets produced 61.3±6.7 ng/ml during an 24-hour incubation period whereas no hHGF was detectable in the L6-WT culture medium (Figure 1C).

**Figure 2:**
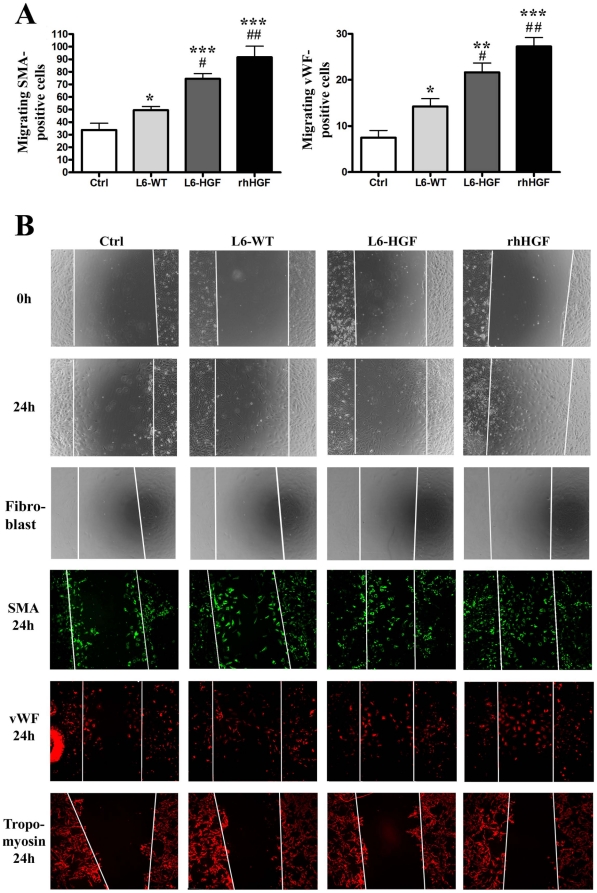
hHGF functionality. Functionality of secreted hHGF was determined *in vitro* by a wound-healing assay with isolated cardiac cells. (A) The number of migrating smooth muscle actin-positive and von Willebrand factor-positive cells into the denuded area 24 hours after wounding was determined from immunofluorescence images. Recombinant human HGF (rhHGF) served as a positive control. (B) Representative images of immunofluorescence stains from all the groups. White lines represent the edges of the denuded area. * p<0.05, ** p<0.01, *** p<0.001 as compared to control; # p<0.05, ## p<0.01 as compared to L6-WT. Data are presented as mean ± SEM.

To ascertain that the hHGF produced by the myoblast sheets was active on primary cells isolated from rat myocardium, we determined by a scratch-wound assay the ability and selectivity of myoblast-secreted hHGF to induce cell migration in these cultures containing cardiomyocytes, endothelial cells, smooth muscle cells, and fibroblasts. To distinguish between cardiac myocyte, smooth muscle cell, and endothelial migration, the cultures were stained for tropomyosin, SMA, and vWF respectively at the end of the experiment. Wounds treated for 24 hours with L6-HGF sheet-conditioned medium showed a significant induction of SMA-positive cell migration with 74.5±4.1 cells as compared to 49.5±3.0 (p<0.05) cells in the L6-WT-conditioned medium-treated, or to 33.8±5.3 (p<0.01) migrating cells in untreated control wounds (Figure 2A). In this assay, recombinant hHGF (100 ng/ml) served as the positive control, and it induced migration of 91.5±8.9 (p<0.001 for control, p<0.01 for L6-WT) SMA-positive cells. Analysis of migration of the vWF-positive cells revealed that the paracrine mediators from L6-HGF myoblast sheets induced a significantly higher number (21.8±2.1) of cells in the denuded area as compared to 7.5±1.6 (p<0.01) for the control group, or 14.0±1.6 (p<0.05) for the L6-WT group. With rhHGF (100 ng/ml), as the control stimulus, the migration induced was 27.3±1.9 cells (p<0.001 for control, p<0.01 for L6-WT) (Figure 2A). We also evaluated the effect of conditioned medium from L6-WT and L6-HGF sheets, with rhHGF again as the positive control on migration of cardiac myocytes staining positive for tropomyosin. In contrast to the effects on migration of SMA-positive smooth muscle and vWF-positive endothelial cells we observed no effect on cardiomyocyte migration of the conditioned media or rhHGF. In the untreated control cultures, we counted 7.3±3.2 cells in the denuded area; the corresponding figure for L6-WT was 6.9±2.6, for L6-HGF 8.0±3.9, and for rhHGF 5.5±3.0. We found no difference in cardiac fibroblast migration between the study groups (Figure 2B).

### Cardiac function

At 4 weeks after ligation of LAD and AMI, we used echocardiography to determine the baseline cardiac function. Subsequent echocardiography measurements were then conducted at 2-week and 4-week time-points after the second surgery and administration of the myoblast sheet therapy to evaluate the effect of L6-WT and L6-HGF therapies on cardiac function. At baseline, a 61 to 63% decline in ejection fraction as compared to the non-infarcted sham group was evident in all LAD-ligated groups (EF %: control 34.56±1.67; L6-WT 32.91±2.38; L6-HGF 34.80±2.19) indicating development of significant heart failure. Two weeks after administration of therapy, we found no differences in ejection fractions between myoblast sheet treatments and the heart failure control group. At 4 weeks, however, the ejection fraction of the control group showed a significant decline against baseline (27.0±1.56, p<0.05 at 4 weeks), while in both the L6-WT (32.68±1.89) and L6-HGF (32.24±1.14) myoblast sheet-treated groups, no such further deterioration of cardiac function was observable and cardiac function was significantly better in both groups as compared to control group (p<0.05 for L6-WT and L6-HGF, Table 1).

**Table 1:** Echocardiography data.

Baseline							
	N	AWTd	PWTd	LVDd	LVDs	FS	EF
**Sham**	**5**	1.50±0.06	1.70±0.18	7.58±0.15	5.32±0.32	30.00±3.17	64.82±4.73
**Control**	**13**	0.55±0.02	1.70±0.06	10.56±0.18	9.22±0.14	13.32±3.66	34.56±1.67
**L6-WT**	**15**	0.59±0.03	1.66±0.09	10.17±0.11	8.89±0.17	12.63±1.08	32.91±2.38
**L6-HGF**	**16**	0.55±0.02	1.53±0.06	10.48±0.16	9.13±0.24	13.51±1.18	34.80±2.19

2 weeks after 2nd surgery							
	N	AWTd	PWTd	LVDd	LVDs	FS	EF
**Sham**	**5**	1.56±0.07	1.90±0.08	8.50±0.13	5.80±0.84	31.69±1.55	67.93±2.07
**Control**	**13**	0.59±0.03	1.67±0.09	10.95±0.17	9.67±0.19	12.38±1.05	31.68±1.86
**L6-WT**	**15**	0.63±0.03	1.55±0.08	10.06±0.17	8.80±0.22	12.64±1.11	33.53±2.04
**L6-HGF**	**16**	0.59±0.03	1.63±0.06	10.75±0.18	9.23±0.23	11.99±0.91	32.93±1.73

4 weeks after 2nd surgery							
	N	AWTd	PWTd	LVDd	LVDs	FS	EF
**Sham**	**5**	1.72±0.07	1.66±0.15	7.72±0.37	4.94±0.19	35.83±1.40	73.44±1.62
**Control**	**13**	0.65±0.02	1.79±0.07	11.76±0.13	10.42±0.20	11.47±1.18	27.01±1.56
**L6-WT**	**15**	0.62±0.04	1.65±0.08	10.71±0.14**	9.37±0.18**	12.54±1.07	32.68±1.89*
**L6-HGF**	**16**	0.63±0.03	1.77±0.07	10.84±0.22*	9.45±0.25*	12.44±0.95	32.24±1.14*

AWTd, anterior wall thickness; PWTd, posterior wall thickness; LVDd, left ventricular diameter, all in diastolic phase. LVDs, left ventricular diameter in systolic phase, all units in mm; FS, fraction shortening (%); EF, ejection fraction (%).

*p<0.05,

**p<0.01.

Moreover, L6-WT and L6-HGF sheet therapies sustained the systolic and diastolic cardiac function by inhibiting left ventricular dilation. At both 2- and 4-week time-points, the L6-WT group had significantly lower LVDs (8.80±0.22 mm, 9.37±0.18, p<0.05 at 2 weeks, p<0.01 at 4 weeks) and LVDd (10.06±0.17 mm, 10.71±0.14, p<0.05 at 2 weeks, p<0.01 at 4 weeks) values than did the control group (LVDs 2 week 9.67±0.19, 4 week 10.42±0.20; LVDd 2 week 10.95±0.17, 4 week 11.76±0.13). For the L6-HGF group, LVDd (10.84±0.22, p<0.05) and LVDs (9.45±0.25, p<0.05) were significantly lower at 4 weeks than in the control group (Table 1).

### *In vivo* angiogenesis after myoblast sheet therapy

We have shown earlier that myoblast sheet therapy induces angiogenesis in chronic heart failure [10]. Now our *in vitro* results in myocardial cell migration indicate that hHGF is a selective and potent inducer of wound healing of endothelial and smooth muscle cells, suggesting that these cells selectively rather than cardiomyocytes could be the target cells of this therapy. Thus both HGF and myoblast sheet therapy harbor proangiogenic potential in the ischemic myocardium [9], [11] and could provide a synergistic therapeutic effect even in the difficult-to-treat setting of chronic heart failure. Based on this background, we evaluated the ability of L6-HGF myoblast sheet therapy to induce angiogenesis *in vivo* in the rat chronic heart failure model.

**Figure 3:**
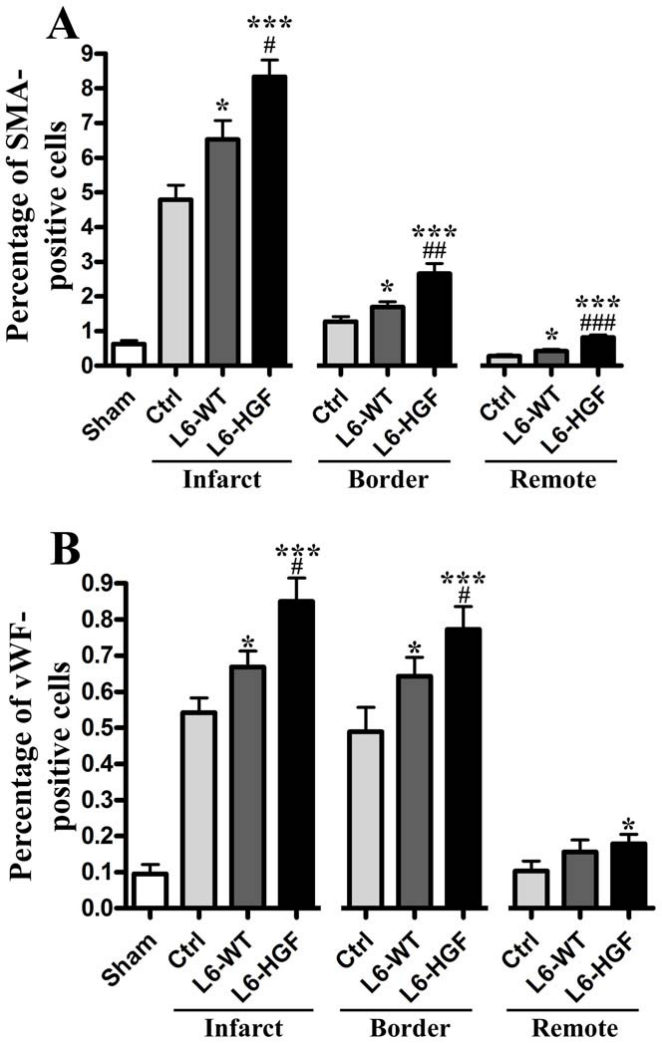
Analysis of angiogenesis *in vivo*. At the study end-point, 4 weeks after administration of therapy, the relative area of smooth muscle actin (SMA) (A) and von Willebrand factor (vWF) (B) –positive cells in the myocardium were determined from sham-operated, control (Ctrl), wild type myoblast sheet therapy (L6-WT) groups, and hHGF-expressing myoblast sheet therapy (L6-HGF) groups. Separate analysis was done of the infarct, border, and remote areas of the myocardium. * p<0.05, *** p<0.001 as compared to control group; # p<0.05, ## p<0.01, ### p<0.001 as compared to L6-WT group. Data are presented as mean ± SEM.

In the post-infarct, non-contractile fibrotic scar, MI induced a strong angiogenic response in the LAD-ligated groups as compared to the sham-operated group as measured by relative amount of SMA- and vWF-positive tissue per whole tissue area. Strikingly, L6-HGF myoblast sheet therapy induced a massive increase in the already high baseline levels of SMA-positive (8.34±0.47%) and vWF-positive (0.86±0.07) tissue as compared to both control (SMA 4.79±0.42, vWF 0.54±0.04, p<0.001 bor both) and L6-WT groups (SMA 6.53±0.54, vWF 0.68±0.03, p<0.05 for both), where as L6-WT therapy had a less pronounced effect (SMA and vWF p<0.05 for control). In the border area of the failing hearts, L6-HGF sheets again induced a highly significant increase of 109% and 59% in SMA-positive (2.66±0.29) and vWF-positive (0.78±0.05) tissue respectively (control SMA 1.27±0.29, vWF 0.49±0.07, p<0.001 for both; L6-WT SMA 1.69±0.15, p<0.01; vWF 0.64±0.05, p<0.05). Also here, L6-WT sheet therapy was significantly less effective in promoting therapeutic angiogenesis, with evidently minor induction of both SMA (p<0.05 for control) and vWF-positive tissue (p<0.05 for control). Furthermore, we found a similar proangiogenic pattern also in the remote area, where L6-HGF therapy was highly effective (SMA 0.82±0.07, p<0.001 for control and L6-WT; vWF 0.18±0.02, p<0.05 for control) to induce neovasculature (Figures 3A and B).

### Analysis of ventricular dilation

**Figure 4:**
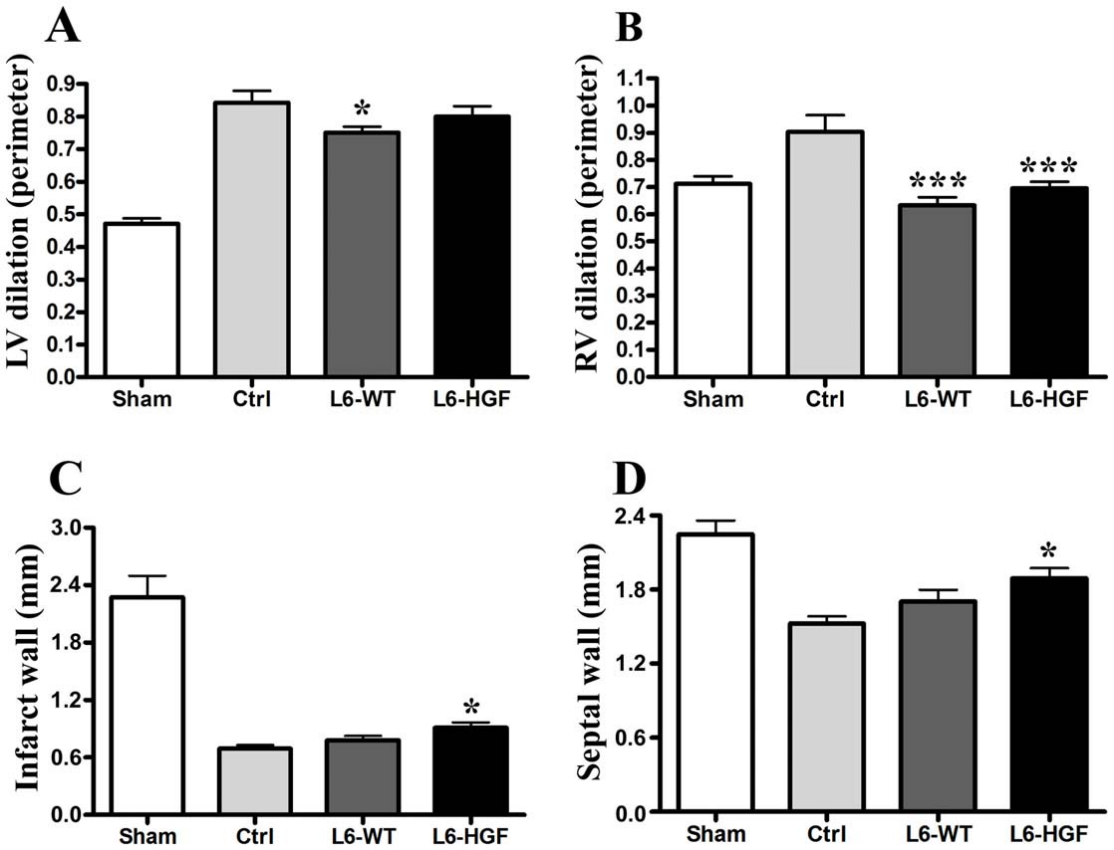
Analysis of cardiac remodeling. Four weeks after wild type (L6-WT) or HGF-expressing (L6-HGF) myoblast sheet therapy, dilation of the left (A) and right (B) ventricles as well as thickness of infarct (C) and septal (D) walls were analyzed from hematoxylin/eosin-stained sections.

For a more detailed analysis of the effect of myoblast sheet therapy on ventricular remodeling, we evaluated from hematoxylin-eosin-stained paraffin-embedded sections left and right ventricle dilation as well as the thickness of the infarct and septal walls. In the left ventricle, the L6-WT group showed significantly less dilation (0.75±0.02) than did the controls (0.87±0.04, p<0.05). In L6-HGF group, however, the effect was less evident (0.81±0.03) and failed to reach statistical significance (Figure 4A). Because, in addition to left ventricular remodeling, MI leads also to remodeling of the right ventricle, we determined whether myoblast sheet therapy could inhibit right ventricular remodeling as well. Interestingly, in the non-infarcted right ventricle, we observed a similar, an even more noticeable effect, because L6-WT myoblast sheet therapy mediated a greater anti-remodeling effect (0.63±0.02, p<0.001 for control) than did the L6-HGF therapy (0.70±0.02, p<0.001 for control) (Figure 4B). Because post-MI remodeling is also associated with ventricular wall thinning [15] and because myoblast sheet therapy induces cell proliferation in the myocardium [9], we measured infarct and septal wall thicknesses to reveal the ablity of myoblast sheet therapy to reduce wall thinning. In both infarct and septal walls, the L6-HGF group (infarct 0.90±0.04, septal 1.91±0.08) had significantly thicker myocardial walls than did the control group (infarct 0.68±0.02, septal 1.53±0.06, p<0.05 for both) (Figure 4 C and D).

### Analysis of fibrosis

Because HGF and myoblast sheet therapy are antifibrotic after acute myocardial infarction [5], [9], we tested whether L6-HGF sheets could reverse or reduce myocardial fibrosis once it already develops, as in the model of MI-induced chronic heart failure. At 4 weeks after therapy, neither L6-WT nor L6-HGF therapy reduced the already developed large fibrotic scar (data not shown).

In the *in vitro* fibrosis assay, human dermal fibroblasts deposited significantly less collagen in presence of L6-HGF conditioned medium (optical density: 0.33±0.02) than in presence of control medium (0.50±0.02, p<0.001) suggesting that hHGF effectively inhibits formation of collagen deposits. However, when collagen was already deposited and the fibrosis inducing factor was absent, hHGF (0.51±0.01) inhibited further accumulation of collagen but was unable to degrade the already existing deposits (0.50±0.004). In rat cardiac fibroblast cultures, similar results were evident as collagen deposition was significantly inhibited but degradation was not induced (Figure S1).

### Gene expression in transfected myoblasts

Because HGF is an autocrine mediator of myoblast proliferation, migration and differentiation [13], and because of the altered morphology of the L6-HGF myoblasts (Figure 1A), we performed a full genome microarray analysis to determine whether constitutive expression of hHGF has autocrine effects on myoblast gene expression. These changes in gene expression might modulate myoblast characteristics as donor cells in myoblast sheet therapy.

The analysis revealed that in L6-HGF myoblast monolayer cultures, 63 genes were upregulated and 85 genes were downregulated by more than 2-fold as compared to L6-WT myoblasts. Differentially expressed genes were associated with migration (Coro1a, Epha3) and proliferation (Efhd1, Grem1) (Table S1). hHGF-expression had no effect on expression of endogenous rat *hgf* or on other major cytokines/growth factors. Further, expression of key regulators of apoptosis remained unchanged, suggesting that hHGF-expression modified neither survival nor paracrine characteristics of the myoblasts. Gene ontology (GO) analysis revealed that the upregulated genes were associated with various biological processes such as wounding and cell differentiation (Table S2).

Importantly, the differences in gene expression were even greater between L6-HGF and L6-WT myoblast sheets than between monolayer cultures. 259 genes were upregulated and 222 downregulated in L6-HGF sheets by over 2-fold as compared to the L6-WT sheets.

### Myoblast apoptosis assay

We evaluated whether hHGF can inhibit myoblast apoptosis in an autocrine manner. HGF did not protect myoblasts against apoptosis induced by different concentrations of staurosporine, suggesting that the L6-HGF sheets do not have a survival benefit (Figure S2).

## Discussion

Our earlier results demonstrated that the effect of myoblast sheet transplantation is mediated mainly by growth factor stimulation of the targeted injured tissue in models of acute and chronic heart failure. We therefore hypothesized that enhancing cell sheet properties by introducing overexpression of a gene associated with angiogenesis, anti-fibrosis, and antiapoptosis could further promote sheet therapy. We evaluated the impact of pleiotropic, cardioprotective *hgf* overexpression in myoblast sheets on their therapeutic efficacy in a rat model of chronic heart failure. This chronic model was chosen because patients receiving open-heart bypass surgery—a procedure during which cell sheet therapy is possible—may suffer multiple infarctions, and their myocardial fibrosis and remodeling have already taken place. Therefore, for a therapy to be effective at this stage, regenerative paracrine stimulation or replacement of tissue would be essential. Employing myoblast sheets as the vehicles for the therapeutic stimulatory paracrine effectors enables higher concentration and longer duration of therapy than with intramyocardial injections of cells or cytokines.

In concert with previous findings demonstrating efficacy of HGF gene therapy [14] promoting myocardial angiogenesis, our data showed—apparently for the first time—that myoblast sheets' proangiogenic potential can be enhanced by HGF therapy. Increased angiogenesis by the hHGF-overexpressing sheets was evident throughout the myocardium, suggesting that both the healthy as well as the injured myocardium responds to this stimulus. Because HGF upregulates VEGF production [15], and mediates antiapoptotic signaling in myoblasts [16], we wanted to rule out the possibility that the stimulation of angiogenesis observed was due to other factors induced by hHGF in the myoblast sheets in an autocrine manner. To elucidate the effect of constitutive hHGF expression on the transplanted myoblasts' gene expression, we performed microarray analysis. Both gene expression and the morphology of the myoblast were strongly altered by constitutive hHGF expression. Microarrays revealed differences in expression of genes related to cell migration and the cell cycle. No major changes were observable in expression of genes associated with apoptosis or paracrine secretion. These data suggest that hHGF does not alter the paracrine profile of myoblast sheets and that the effects observed *in vivo* are due to hHGF alone or to hHGF-stimulated target tissue responses.

The major mechanism behind the therapeutic action of myoblast sheet transplantation is paracrine stimulation of the injured myocardium. Although skeletal myoblasts produce several factors [17], the majority of these are positive regulators of angiogenesis. In our study using the rat chronic heart failure model, we demonstrated that endothelial cell responses to myoblast sheet-derived paracrine factors are mediated by VEGF-A and PlGF [9]. Induction of VEGF is an early, yet persistant response to MI. In contrast, HGF is induced in MI only after its acute stage. HGF exerts its mitogenic, motogenic, and morphogenic actions in a complimentary and synergistic fashion, yet one distinct from that of VEGF [18]. Studies in acute MI models have shown the beneficial functional effect of HGF therapy to be mediated through induction of angiogenesis [11], reduction of fibrosis [5], and contribution to recruitment of stem cells from the bone marrow [19] – effects associated with myocardial regeneration.

Our findings, however, are in direct contrast to findings of functional improvement after HGF therapy in acute MI models. This can be attributed to differences in experimental settings. We previously showed, using this chronic heart failure model, that an overall enhancement of myoblast sheet functionality and survival by antiapoptotic Bcl-2 was associated with improved left ventricular function. The fact that the introduction of a single cytokine could not reproduce this effect despite its proangiogenic ability suggests that in this chronic setting, functional improvement and therapeutic angiogenesis may be specific and separate components and may thus be uncoupled. Furthermore, our results imply that enhancing the production of a single paracrine mediator is not a viable therapy option when designing modifications of myoblast sheet therapy. The fact that LV function failed to improve was not associated with inability of the human HGF to induce motility of rat cardiac cells. In fact, those factors produced by L6-HGF sheets were superior to ones produced by L6-WT sheets to promote vWF and SMA-positive cell, but not fibroblast, migration.

Others have shown HGF to suppress the development of fibrosis after MI [5]. The ability of HGF to reverse existing fibrosis when it has developed, weeks after MI, has not been reported. Our data suggests that once the infarct scar is fully developed, HGF alone is insufficient to reduce that scar. In counteracting fibrosis, HGF promotes extracellular matrix remodeling via upregulation of proteolytic factors such as matrix metalloprotease 1 [20]. Furthermore, HGF downregulates expression of TGF-B, a key regulator of fibrosis that promotes matrix deposition by myofibroblasts [21]. In the current model, myocardial fibrosis was fully developed before therapy administration. Expression of acute profibrotic factors such as TGF-B1 and TGF-B2 had therefore receded [22]. Although expression of the third TGF-B isoform, TGF-B3, may be sustained for a longer period, HGF seems to be unable to modulate already established fibrosis in a manner providing any functional benefit.

We conclude that, after an MI, HGF supplementation from myoblast sheets is an effective strategy to enhance angiogenesis in the chronically failing fibrotic myocardium. In contrast to several other reports linking myocardial angiogenesis to enhanced cardiac performance, our data do not support such a direct association.

## Supporting Information

Figure S1 **Analysis of the antifibrotic effect of hHGF** ***in vitro*** **.** L-ascorbic acid 2-phosphate induced collagen deposition in control (Ctrl, standard culture medium) cultures as compared to untreated baseline in human dermal and rat cardiac fibroblasts. When collagen was induced in presence of hHGF from L6 myoblasts (L6-HGF), deposition was significantly inhibited (upper panel). When L-ascorbic acid 2-phosphate-induced collagen was already deposited for 7 days (baseline) and then removed from culture, L6-HGF paracrine factors inhibited further deposition as compared to control (standard culture medium) but could not reduce existing deposits in either dermal or cardiac fibroblasts (lower panel). *** p<0.001 as compared to control; ### p<0.001 as compared to baseline.
(EPS)

Figure S2 **Evaluation of apoptosis in myoblasts.** Wild type (L6-WT) or hHGF-expressing (L6-HGF) myoblasts were treated with indicated concentrations of staurosporine for 24 hours to induce apoptosis. hHGF-expression did not significantly affect myoblast apoptosis in an autocrine manner.
(EPS)

Table S1 **Differential gene expression by hHGF.** Ten most upregulated and downregulated genes in L6-HGF myoblasts as compared to L6-WT myoblasts in monolayer culture.
(XLSX)

Table S2 **Gene ontology (GO) categories.** **GO** groups significantly associated with the differentially expressed genes in L6-HGF myoblasts as compared to L6-WT myoblasts.
(XLSX)
